# Supplementary material for: BioAIEgens derived from rosin: how does molecular motion affect their photophysical processes in solid state?
Source: Nat Commun. 2021 Mar 19;12:1773. doi: 10.1038/s41467-021-22061-y (PMC7979920; doi:10.1038/s41467-021-22061-y)
Supplement: Supplementary file 1 — Supplementary Information [file 41467_2021_22061_MOESM1_ESM.pdf]

Supplementary Information for

BioAIEgens Derived from Rosin: How Does Molecular Motion Affect Their Photophysical Processes in Solid State?

Cai et al

Content

**Experimental Section**	S1-S2
Synthesis and Characterizations	S1-S2
**Figures and Tables**	S3-S18
Supplementary Figure 1	S3
Supplementary Figure 2	S3
Supplementary Figure 3	S4
Supplementary Figure 4	S4
Supplementary Figure 5	S5
Supplementary Figure 6	S5
Supplementary Figure 7	S6
Supplementary Figure 8	S6
Supplementary Figure 9	S7
Supplementary Figure 10	S7
Supplementary Figure 11	S8
Supplementary Figure 12	S9
Supplementary Figure 13	S9
Supplementary Figure 14	S10
Supplementary Figure 15	S10
Supplementary Figure 16	S11
Supplementary Figure 17	S11
Supplementary Table 1	S12
Supplementary Figure 18	S13
Supplementary Figure 19	S13
Supplementary Figure 20	S14
Supplementary Figure 21	S15
Supplementary Figure 22	S15
Supplementary Figure 23	S15
Supplementary Figure 24	S16
Supplementary Figure 25	S16
Supplementary Figure 26	S17
Supplementary Figure 27	S17
**References**	S18

Experimental Section

Synthesis and Characterization

AB-SA: Salicylaldehyde (31.3 μL, 0.3 mmol) was slowly added to a vigorously stirred solution of 2-bromoaniline (51.6 mg, 0.3 mmol) in 3 mL of MeOH. After 10 min of stirring at room temperature, the resulting mixture was filtered and dried in air to produce a yellow solid, which was then used for recrystallization in an ethanol solution. A yellow crystalline product was obtained with a yield of around 65%. Due to the rough crystallographic data found in the literature,[1] its singles crystals suitable for X-ray crystallographic measurements were obtained by slow evaporation from an EtOH solution, which were re-measured, resolved, and refined, in order to meet the discussion requirements in this work. mp: 85-86 °C; ^{1}H NMR (600 MHz, $CDCl_3$): δ 13.03 (s, 1H), 8.60 (s, 1H), 7.68 (dd, J = 8.1, 1.2 Hz, 1H), 7.42-7.36 (m, 3H), 7.23 (dd, J = 7.8, 1.2 Hz, 1H), 7.16-7.13 (m, 1H), 7.07-7.05 (m, 1H), 6.97-6.94 (m, 1H).

DAMB-SA: A solution of salicylaldehyde (15.6 μL, 0.15 mmol) in 5 mL of EtOH was slowly added to a vigorously stirred solution of DAMBA (54.9 mg, 0.15 mmol) in 5 mL of EtOH. The reaction mixture was refluxed at 90 °C for 10 h and cooled to room temperature afterwards. The obtained precipitates were then used for recrystallization in an ethanol solution, producing plenty of fine yellow crystals with a crystalline yield of around 75%. Yellow block-shaped single crystals suitable for X-ray crystallographic measurements were obtained by slow evaporation from an EtOH solution. mp: 156-157 °C; ^{1}H NMR (600 MHz, $CDCl_3$): δ 13.24 (s, 1H), 8.56 (s, 1H), 7.51 (s, 1H), 7.39-7.36 (m, 2H), 7.03 (dd, 1H), 6.94-6.91 (m, 2H), 2.90-2.88 (m, 2H), 2.27 (m, 1H), 2.20 (m, 1H), 1.87-1.74 (m, 4H), 1.68-1.66 (m, 4H), 1.52-1.45 (m, 2H), 1.28 (s, 3H), 1.22 (s, 3H); ^{13}C NMR (150 MHz, $CDCl_3$): δ 178.9, 162.3, 161.3, 150.3, 143.9, 135.8, 133.5, 132.4, 129.2, 119.2, 119.16, 119.13, 117.5, 117.4, 52.1, 47.6, 44.6, 38.0, 37.4, 36.7, 29.7, 25.1, 21.4, 18.5, 16.6; HRMS (MALDI-TOF, m/z): $[M+H]^+$ calcd for $C_{25}H_{29}BrNO_3$: 470.1325; found 470.1330.

DAMB-SAB: DAMB-SAB was prepared according to the above synthesis procedures of DAMB-SA, with 3,5-di-tert-butyl-salicylaldehyde instead of salicylaldehyde. The reaction mixture was refluxed at 100 °C over night and cooled to room temperature afterwards. The obtained precipitates were then used for recrystallization in a CH_3CN/CH_2Cl_2 (5:6, v/v) solution, producing plenty of yellow block-shaped single crystals that were suitable for X-ray crystallographic measurements (crystalline yield of around 70%). mp: 104-105 °C; ^{1}H NMR (600 MHz, $CDCl_3$): δ 13.45 (s, 1H), 8.56 (s, 1H), 7.51 (s, 1H), 7.47 (d, J = 2.4 Hz, 1H), 7.22 (d, J = 2.4 Hz,1H), 6.89 (s, 1H), 3.69 (s, 3H), 2.90-2.87 (m, 2H), 2.28 (m, 1H), 2.21 (m, 1H), 1.88-1.74 (m, 4H), 1.69-1.68 (m, 1H), 1.55-1.52 (m, 2H), 1.49 (s, 9H), 1.34 (s, 9H), 1.30 (s, 3H), 1.24 (s, 3H); ^{13}C NMR (150 MHz, $CDCl_3$): δ 179.0, 163.9, 158.6, 149.8, 144.6, 140.7, 137.4, 135.8, 129.2, 128.5, 127.0, 119.5, 118.4, 117.1, 52.1, 47.7, 44.8, 38.1, 37.4, 36.8, 35.3, 34.3, 31.6, 29.7, 29.6, 25.2, 21.6, 18.6, 16.7; HRMS (MALDI-TOF, m/z): $[M]^+$ calcd for $C_{33}H_{44}BrNO_3$: 581.2505; found 581.2489.

DAMB-SAN: DAMB-SAN was prepared according to the above synthesis procedures of DAMB-SA, with 5-nitro-salicylaldehyde instead of salicylaldehyde. The reaction mixture was refluxed at 85 °C for 3 h and cooled to room temperature afterwards. The obtained precipitates were then used for recrystallization in an ethanol solution, producing plenty of yellow crystals with a crystalline yield of around 65%. Yellow block-shaped single crystals suitable for X-ray crystallographic measurements were obtained by slow evaporation in a EtOH/CH_2Cl_2 (1:1, v/v) mixture. mp: 216-217 ºC; ^{1}H NMR (600 MHz, $CDCl_3$): δ 14.31 (s, 1H), 8.67 (s, 1H), 8.40 (d, J = 2.7 Hz 1H), 8.26 (dd, J = 9.2, 2.7 Hz, 1H), 7.54 (s, 1H), 7.10 (d, J = 9.2 Hz, 1H), 6.99 (s, 1H), 3.69 (s, 3H), 2.93-2.89 (m, 2H), 2.28 (m, 1H), 2.20 (m, 1H), 1.88-1.74 (m, 4H), 1.70-1.68 (m, 1H), 1.53-1.48 (m, 2H), 1.29 (s, 3H), 1.23 (s, 3H); ^{13}C NMR (150 MHz, $CDCl_3$): δ 178.9, 166.8, 160.2, 151.6, 142.4, 140.1, 136.2, 129.6, 128.6, 128.5, 119.2, 118.6, 118.3, 117.7, 52.2, 47.6, 44.6, 38.0, 37.6, 36.7, 29.7, 25.1, 21.4, 18.5, 16.6; HRMS (MALDI-TOF, m/z): $[M+H]^+$ calcd for $C_{25}H_{28}BrN_2O_5$: 515.1176; found 515.1166.

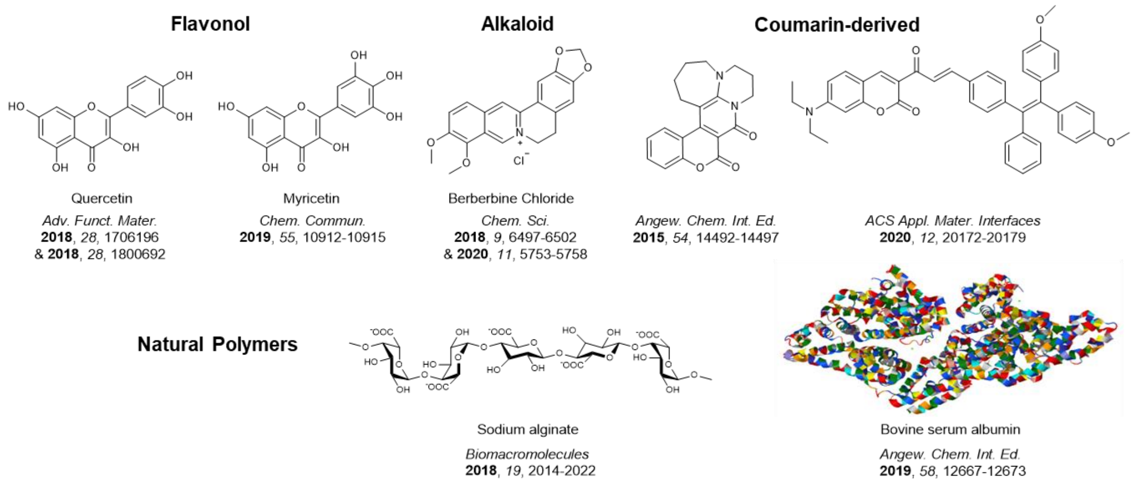

Supplementary Figure 1. A summary of the reported BioAIEgens (BioAIEgens represent AIEgens or AIE-active nano agents obtained from natural resources including natural products or derivatives by modifying natural products). Single crystal structure of bovine serum albumin drawn by amino acid was adapted from Ref. [2].

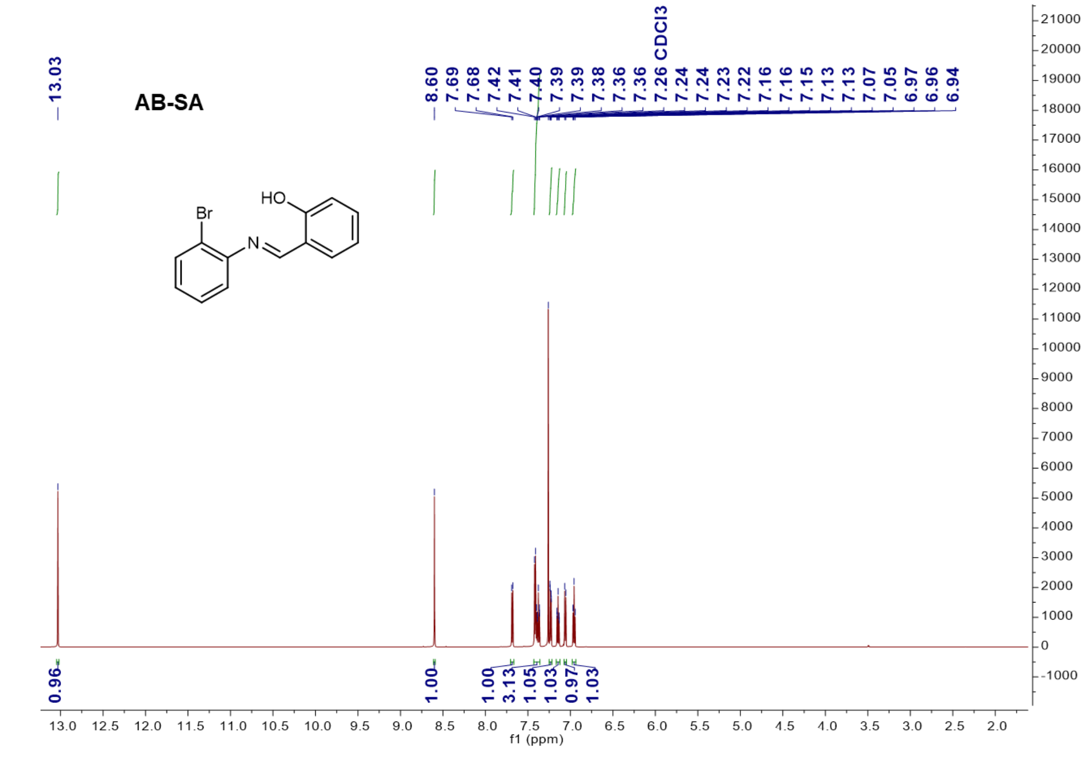

Supplementary Figure 2. ^{1}H NMR spectrum of AB-SA.

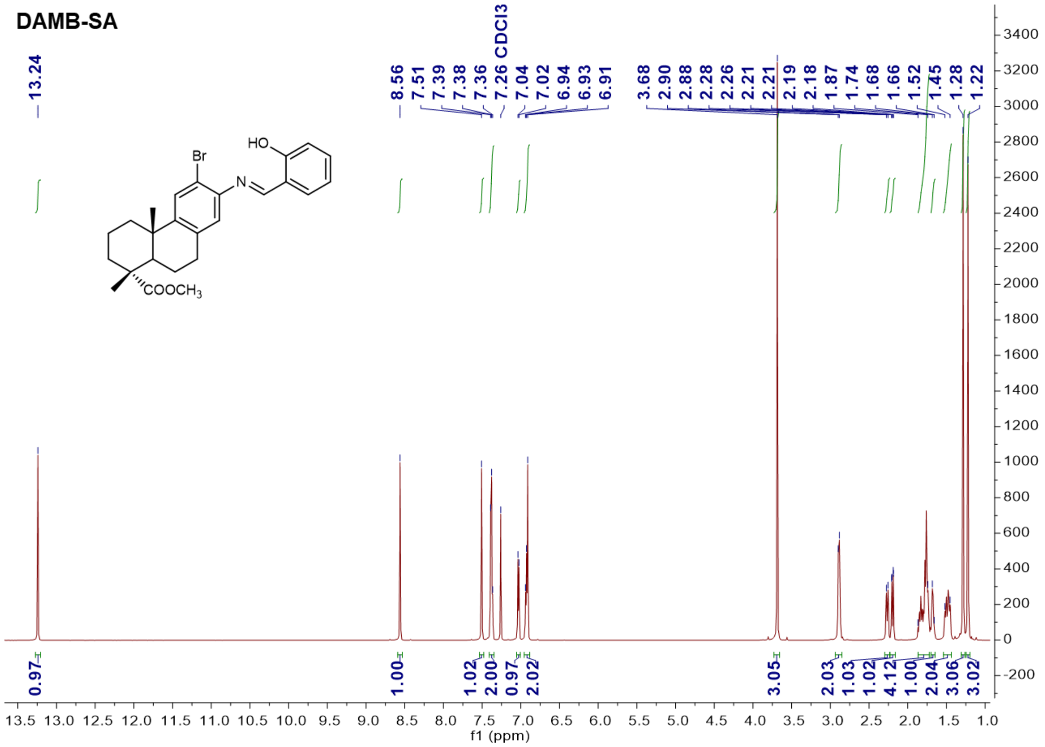

Supplementary Figure 3. ^{1}H NMR spectrum of DAMB-SA.

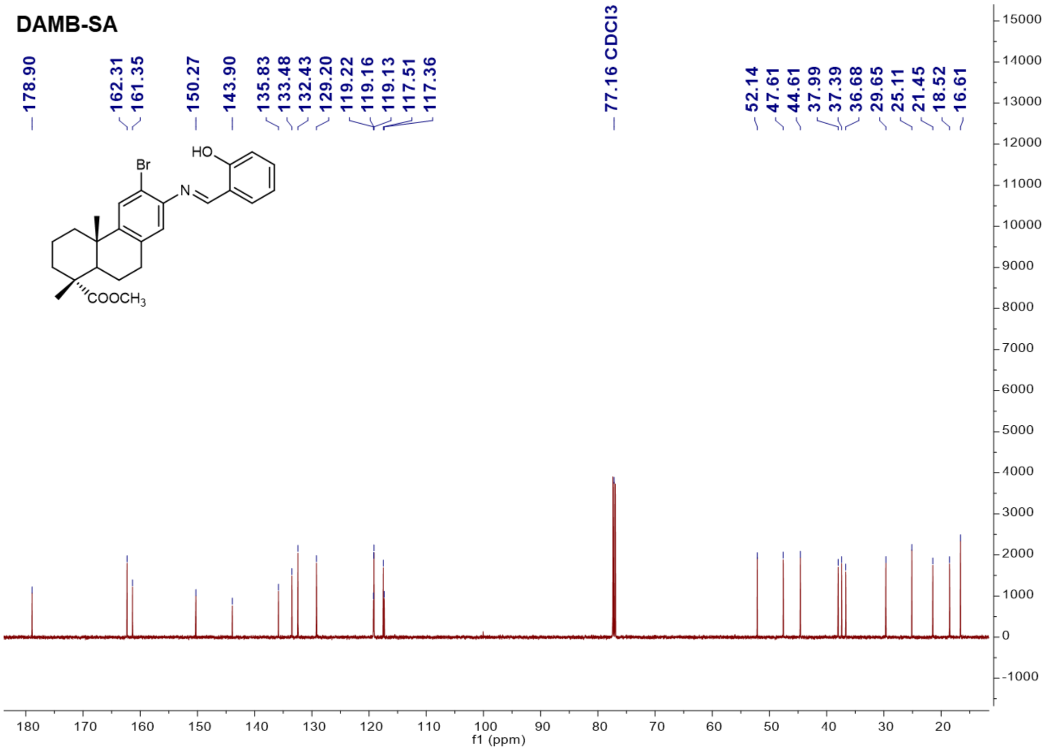

Supplementary Figure 4. ^{13}C NMR spectrum of DAMB-SA.

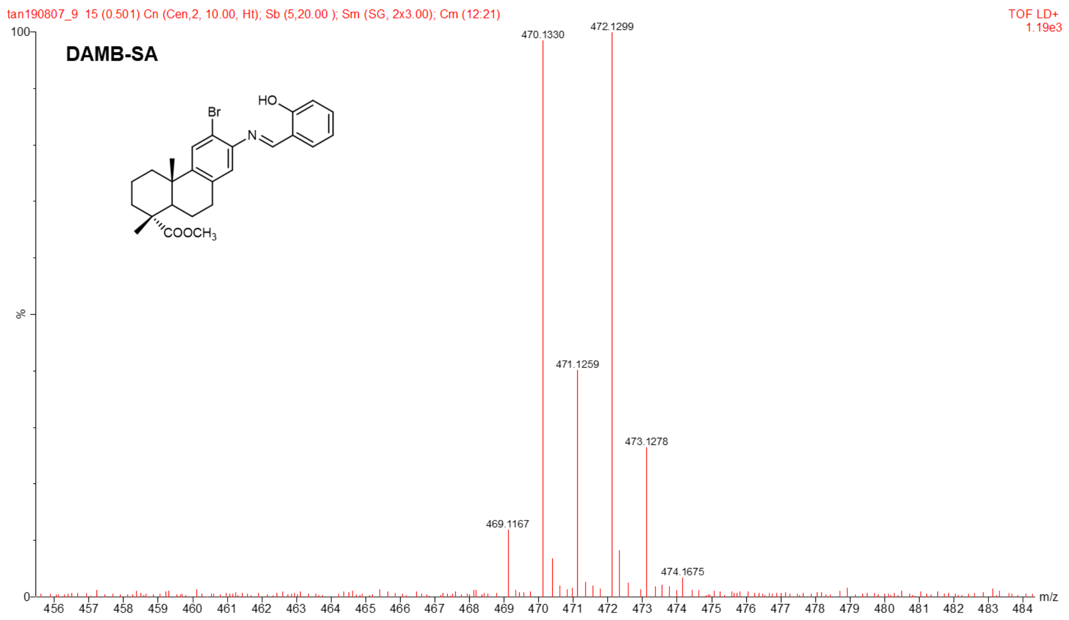

Supplementary Figure 5. High resolution mass spectrum of DAMB-SA.

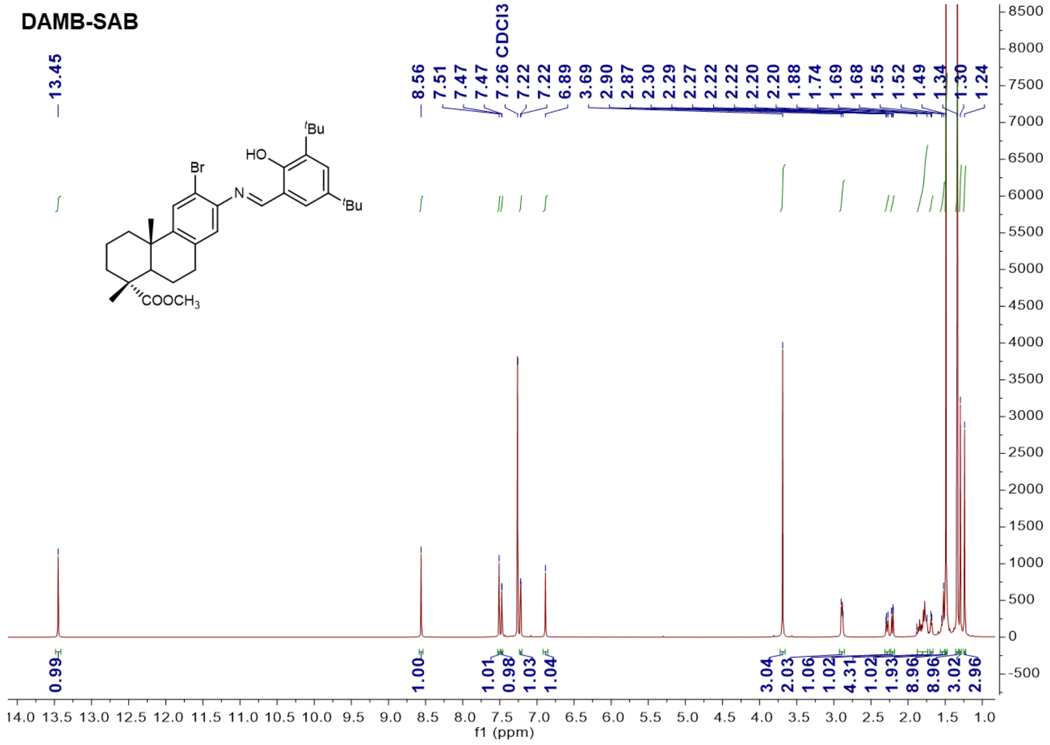

Supplementary Figure 6. ^{1}H NMR spectrum of DAMB-SAB.

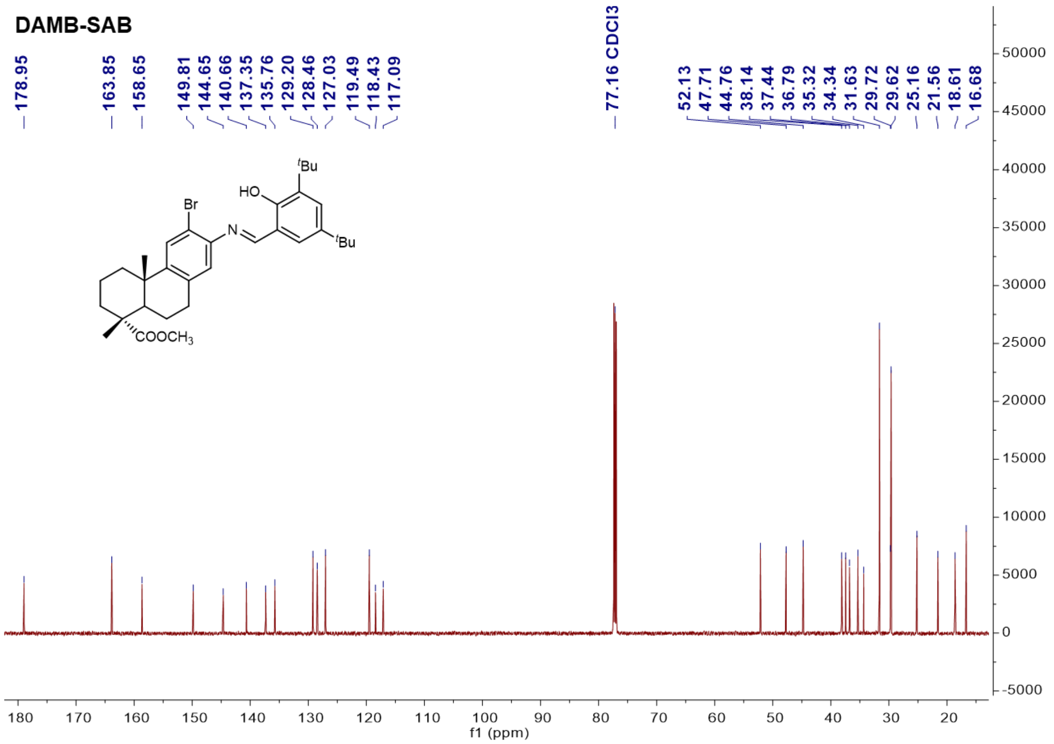

Supplementary Figure 7. ^{13}C NMR spectrum of DAMB-SAB.

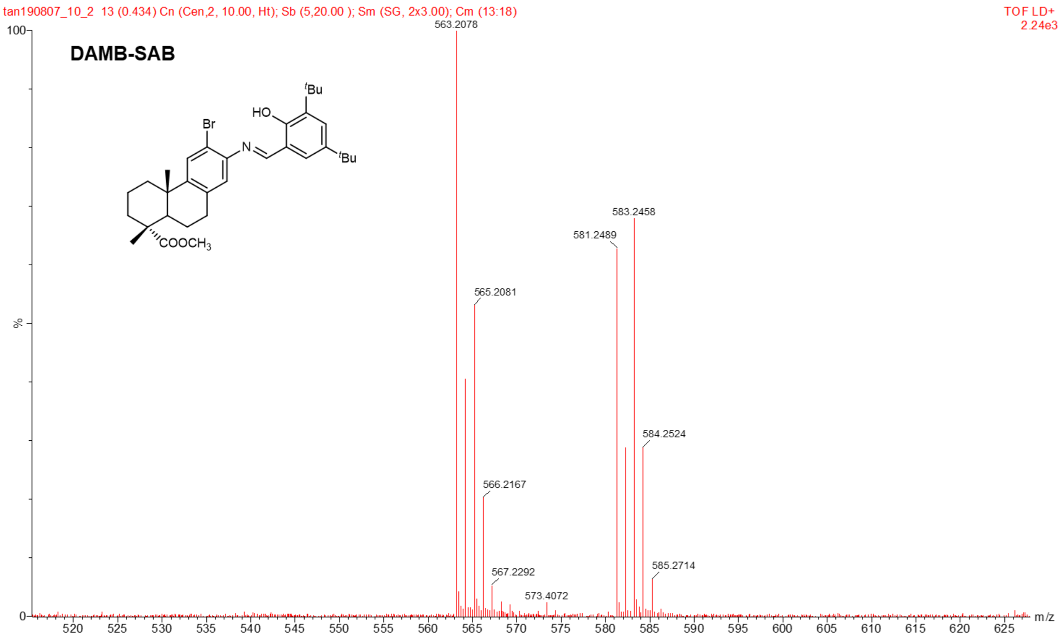

Supplementary Figure 8. High resolution mass spectrum of DAMB-SAB.

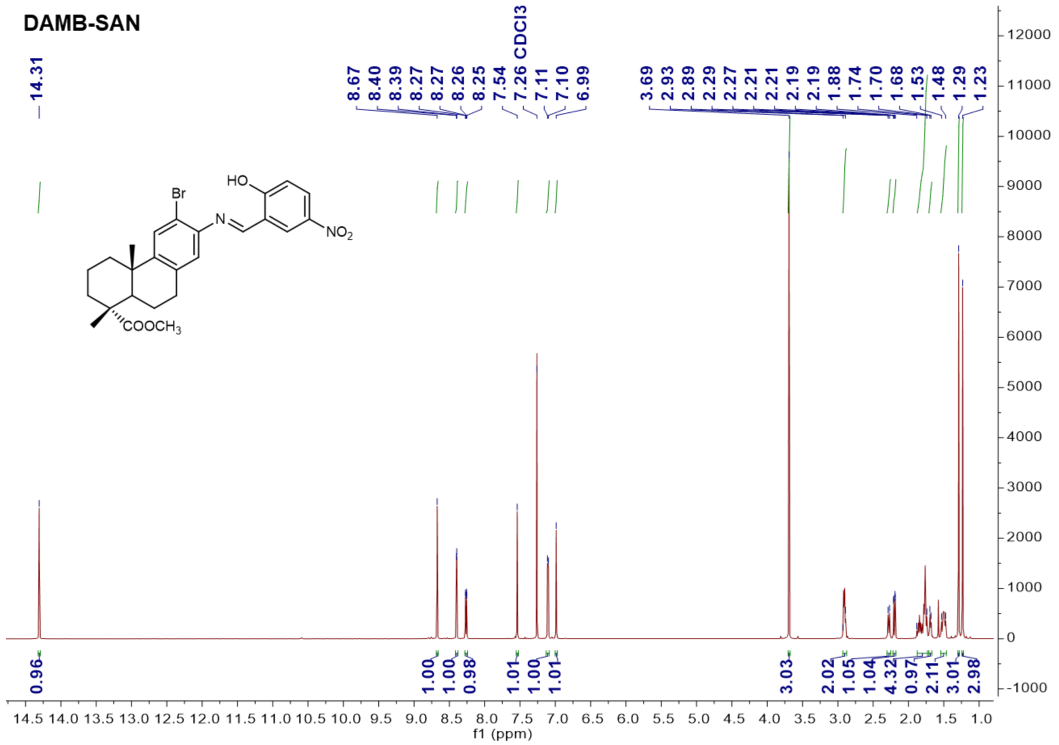

Supplementary Figure 9. ^{1}H NMR spectrum of DAMB-SAN.

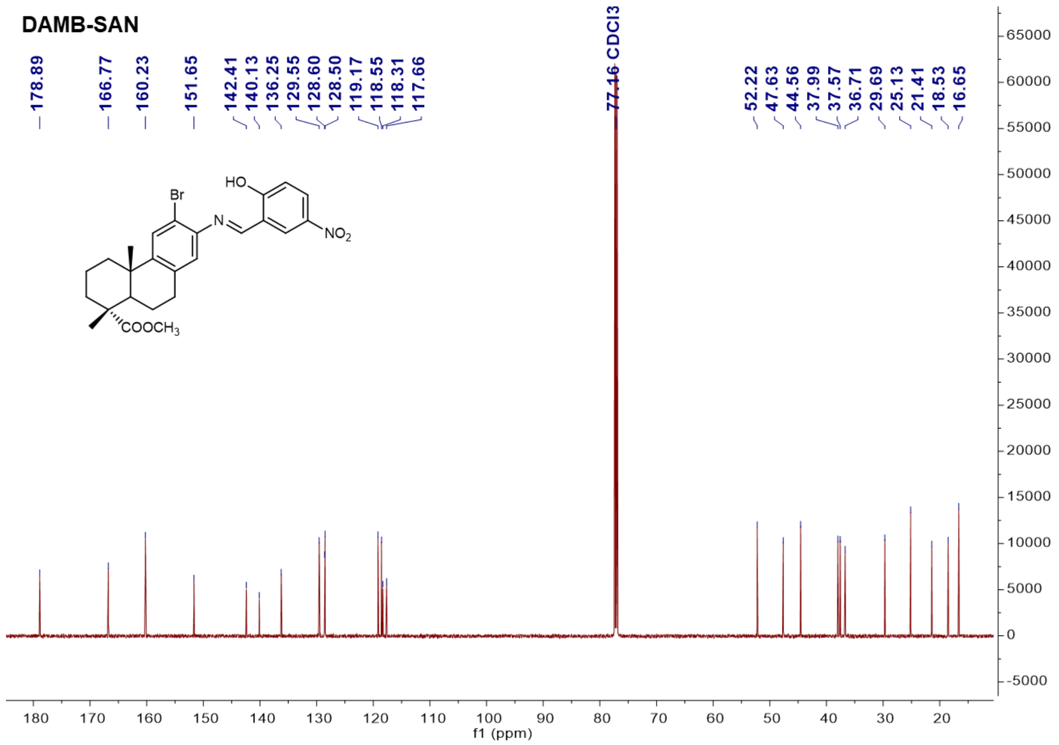

Supplementary Figure 10. ^{13}C NMR spectrum of DAMB-SAN.

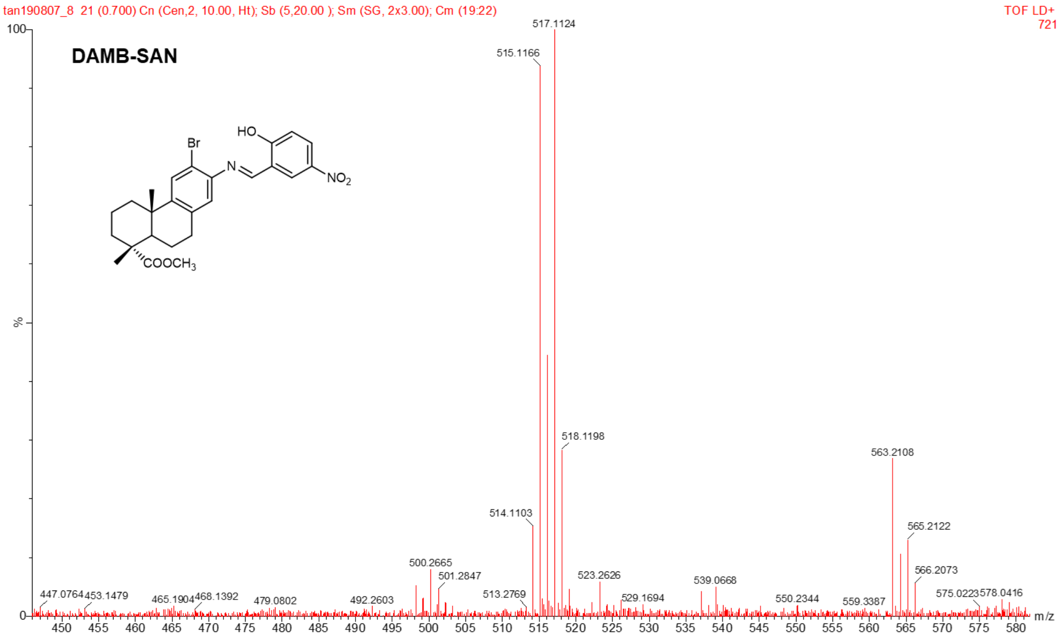

Supplementary Figure 11. High resolution mass spectrum of DAMB-SAN.

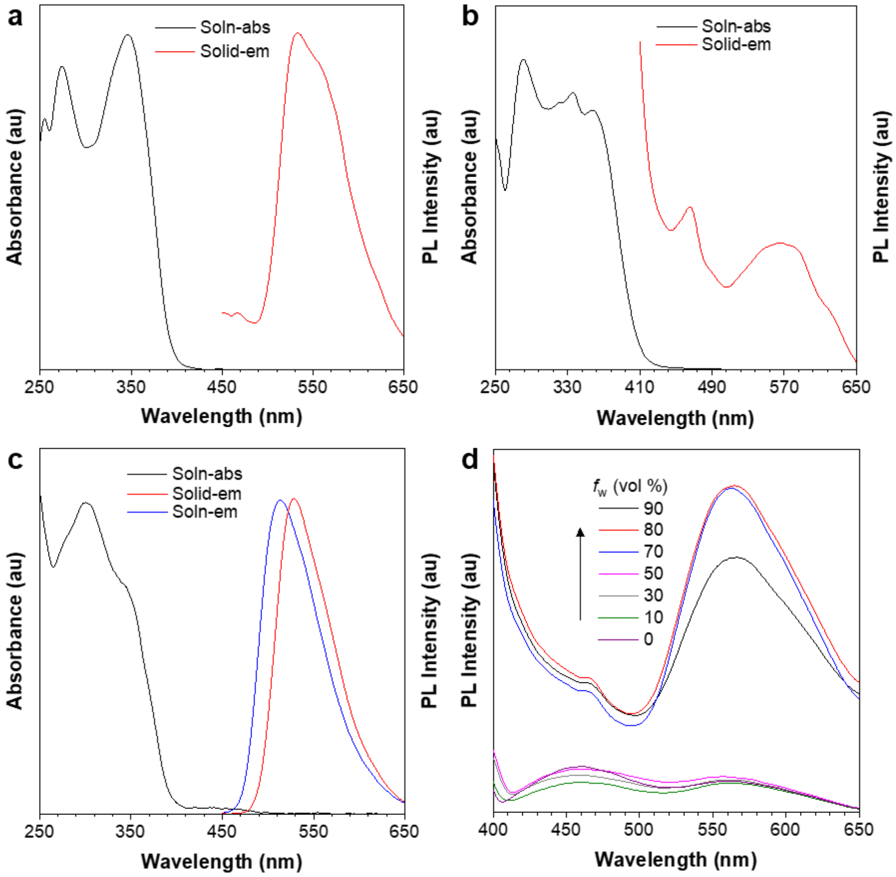

Supplementary Figure 12. a, b and c Absorption and PL spectra of (**a**) DAMB-SA, (**b**) DAMB-SAB and (**c**) DAMB-SAN in dilute ACN solution (20 μM) and as solid. **d** PL spectra of DAMB-SAB in ACN/H_2O mixtures with different water fractions (f_w). Concentration: 20 μM; λ_{ex}: 350 nm.

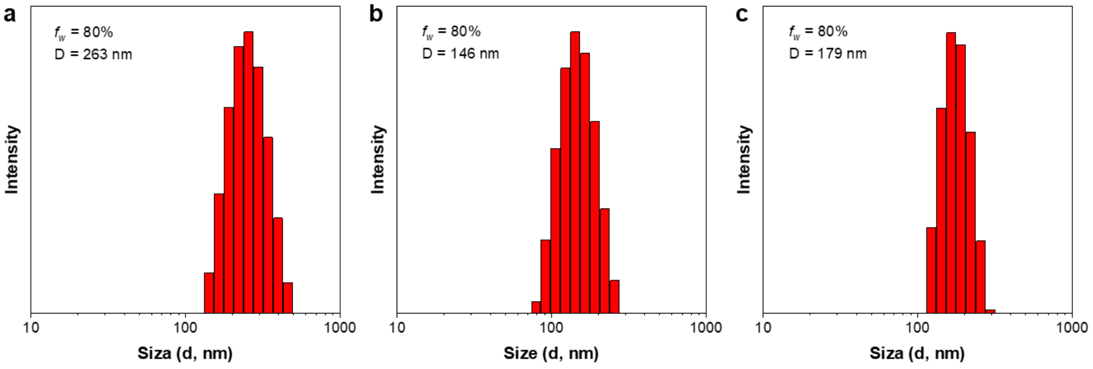

Supplementary Figure 13. DLS results of (**a**) DAMB-SA, (**b**) DAMB-SAB, and (**c**) DAMB-SAN in ACN/H_2O mixtures with f_w = 80% (20 μM), respectively.

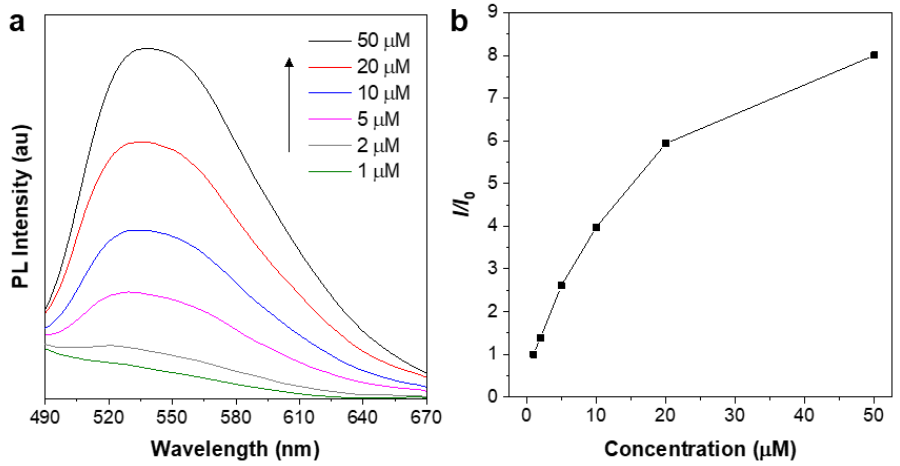

Supplementary Figure 14. a PL spectra of DAMB-SA in ACN solution with different concentrations. λ_{ex}: 350 nm. **b** The plots of the emission intensity at the maximum versus the concentration of DAMB-SA in ACN. I_0 = PL intensity of DAMB-SA in ACN solution (1 μM).

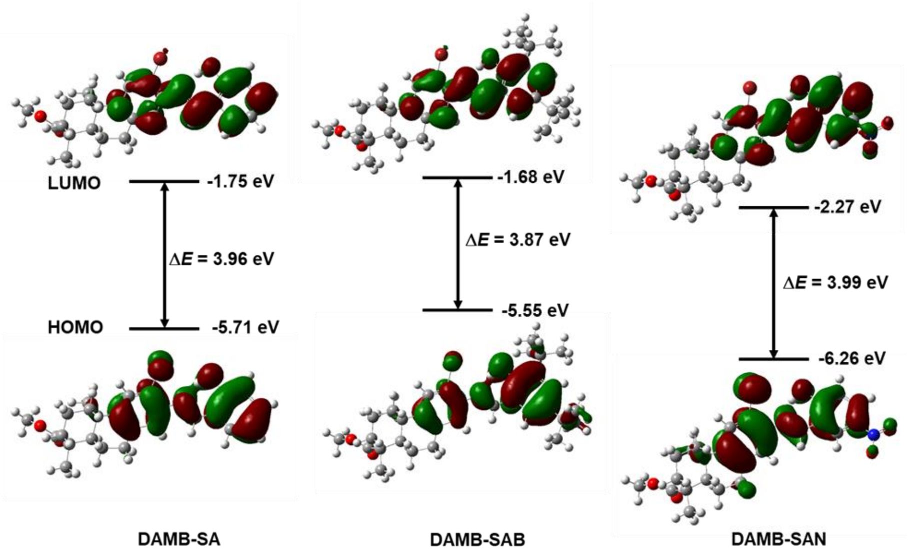

Supplementary Figure 15. Frontier molecular orbitals of DAMB-SA, DAMB-SAB, and DAMB-SAN and their corresponding HOMO and LUMO energies.

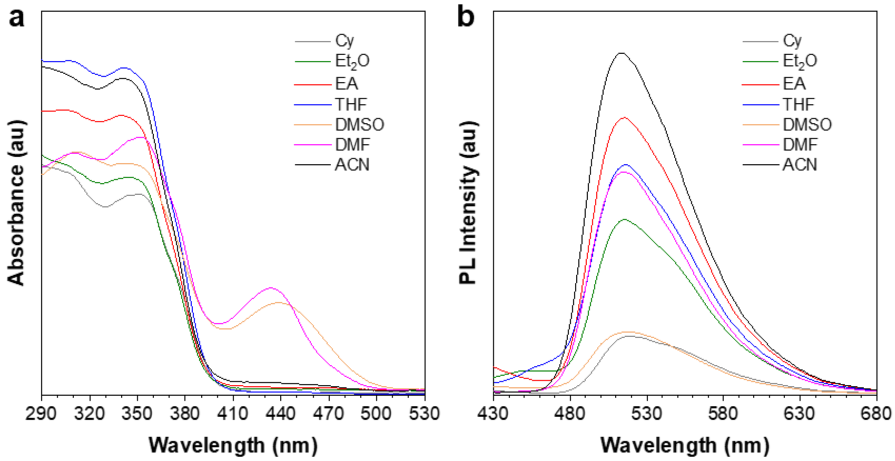

Supplementary Figure 16. a Absorption and (**b**) PL spectra of DAMB-SAN in solvents with different polarities. Concentration: 20 μM. The absorption maximum of each solution was chosen as its excitation wavelength.

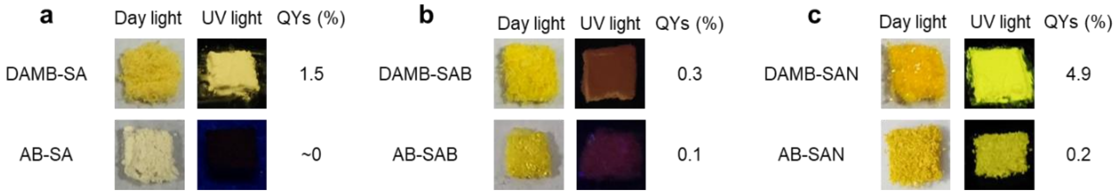

Supplementary Figure 17. Figures and QYs of (**a**) DAMB-SA and AB-SA, (**b**) DAMB-SAB and AB-SAB, and (**c**) DAMB-SAN and AB-SAN under Day light and UV light, respectively.

Supplementary Table 1. Crystallographic data for compounds AB-SA, DAMB-SA, DAMB-SAB, and DAMB-SAN.

	**AB-SA**	**DAMB-SA**	**DAMB-SAB**	**DAMB-SAN**
empirical formula	$C_{13}H_{10}BrNO$	$C_{25}H_{28}BrNO_3$	$C_{33}H_{44}BrNO_3$	$C_{25}H_{27}BrN_2O_5$
M_r	276.12	470.38	582.59	515.39
cryst syst	orthorhombic	monoclinic	orthorhombic	monoclinic
space group	$P2_12_12_1$	$P2_1$	$P2_12_12_1$	$P2_1$
a (Å)	7.0763(2)	7.2788(4)	12.6705(8)	14.8914(4)
b (Å)	12.2610(3)	24.9017(14)	14.387(1)	6.7738(2)
c (Å)	13.3259(3)	12.2127(7)	17.0340(11)	23.3672(6)
α (º)	90	90	90	90
β (º)	90	94.650(2)	90	104.627(2)
γ (º)	90	90	90	90
V (Å^3)	1156.19(5)	2206.3(2)	3105.1(4)	2280.69(11)
Z	4	4	4	4
ρ_c (g cm^{-3})	1.586	1.416	1.246	1.501
F (000)	552.0	976.0	1232.0	1064.0
T (K)	293	273	296	100
μ (mm^{-1})	4.654	1.890	1.356	2.794
data / restraints / parameters	2051 / 0 / 146	8601 / 3 / 549	7118 / 30 / 353	6528 / 1 / 604
GOF (F^2)	1.017	1.015	1.082	1.051
$R1^a$, $wR2^b$ ($I > 2\sigma(I)$)	0.0262, 0.0670	0.0455, 0.0777	0.0580, 0.1247	0.0472, 0.1222
R_{int}	0.0220	0.0521	0.0510	0.0325

^{a} $R_1 = \Sigma(||F_o| - |F_c||)/\Sigma|F_o|$; ^{b} $wR_2 = \{\Sigma[w(F_o^2 - F_c^2)^2]/\Sigma[w(F_o^2)^2]\}^{1/2}$

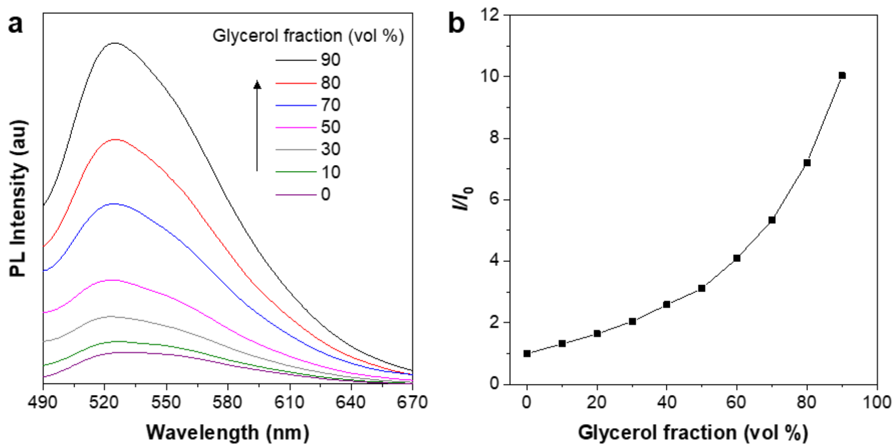

Supplementary Figure 18. a PL spectra of DAMB-SA in EtOH/glycerol mixtures with different fractions of glycerol. Concentration: 20 μM; λ_{ex}: 350 nm. **b** The plots of the emission intensity at the maximum versus the composition of the glycerol mixture of DAMB-SA. I_0 = PL intensity in pure EtOH. Concentration: 20 μM.

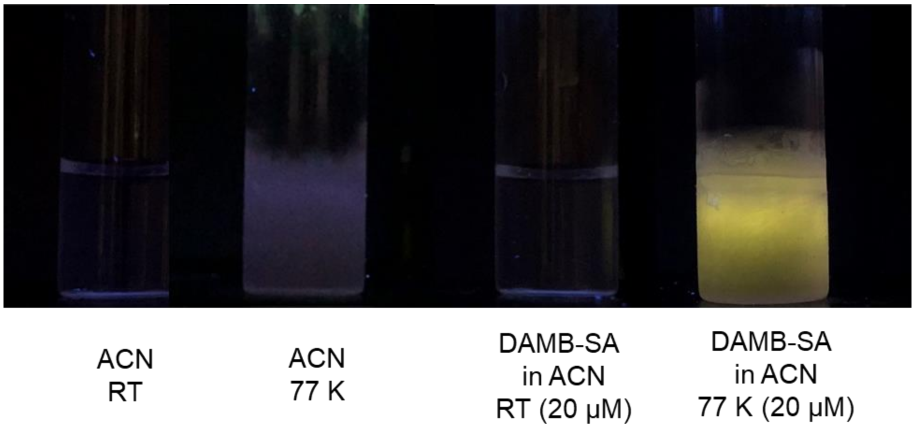

Supplementary Figure 19. Fluorescence images of sole ACN and DAMB-SA solution in ACN at RT and 77 K, respectively. λ_{ex}: 365 nm.

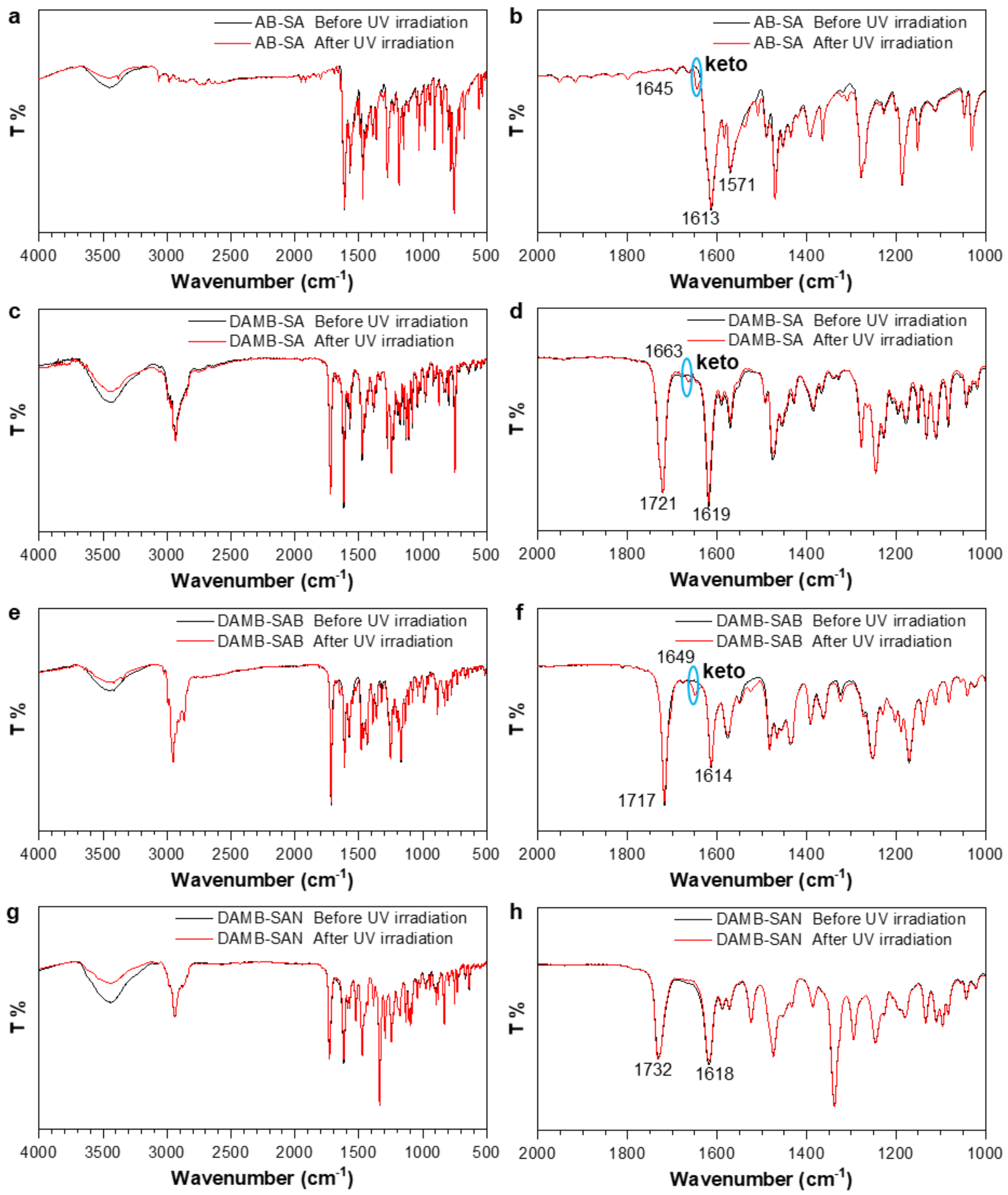

Supplementary Figure 20. IR spectra of (**a** and **b**) AB-SA, (**c** and **d**) DAMB-SA, (**e** and **f**) DAMB-SAB, and (**g** and **h**) DAMB-SAN before and after UV irradiation at 365 nm (left: full spectra; right: spectra in the wavenumber range of 2000 to 1000 cm^{-1}).

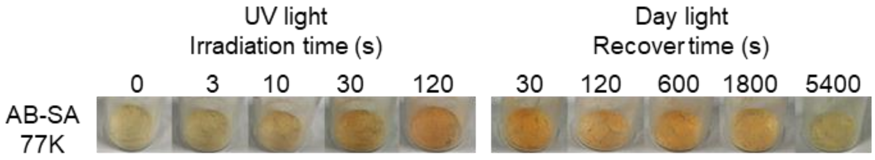

Supplementary Figure S21. Photochromic images of AB-SA (77 K) upon UV and Day light irradiation.

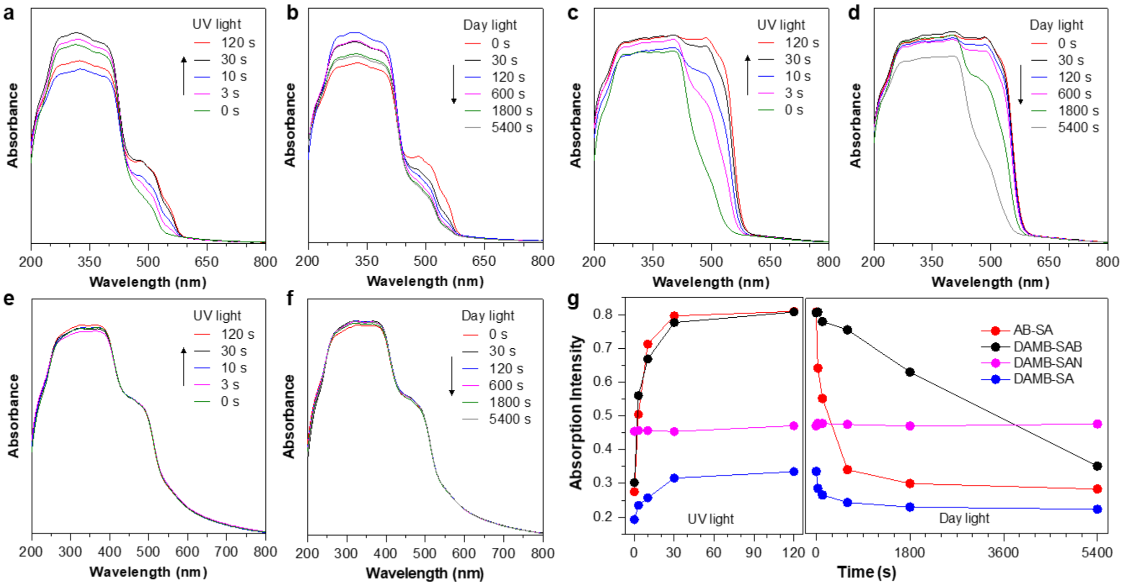

Supplementary Figure 22. a-f UV-DRS spectra of (**a** and **b**) DAMB-SA, (**c** and **d**) DAMB-SAB, and (**e** and **f**) DAMB-SAN for their respective reversible photochromic processes (0 s to 120 s to 5400 s). **g** The plots of the visible absorption maximum of AB-SA (469 nm), DAMB-SA (480 nm), DAMB-SAB (485 nm), and DAMB-SAN (480 nm) versus the irradiation and recover time, respectively.

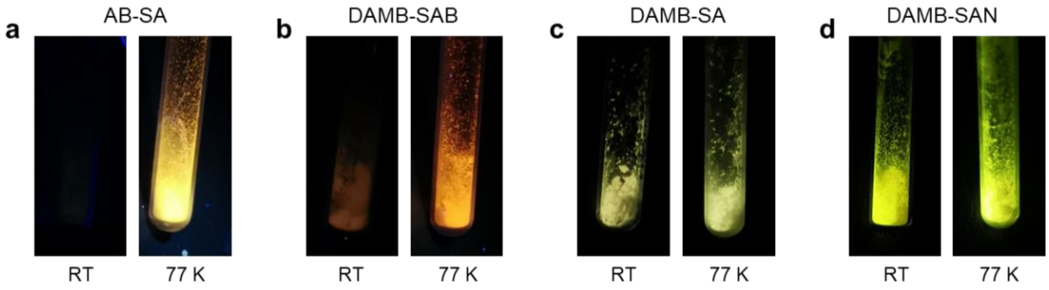

Supplementary Figure 23. Fluorescence figures of (**a**) AB-SA, (**b**) DAMB-SAB, (**c**) DAMB-SA, and (**d**) DAMB-SAN at RT and 77 K.

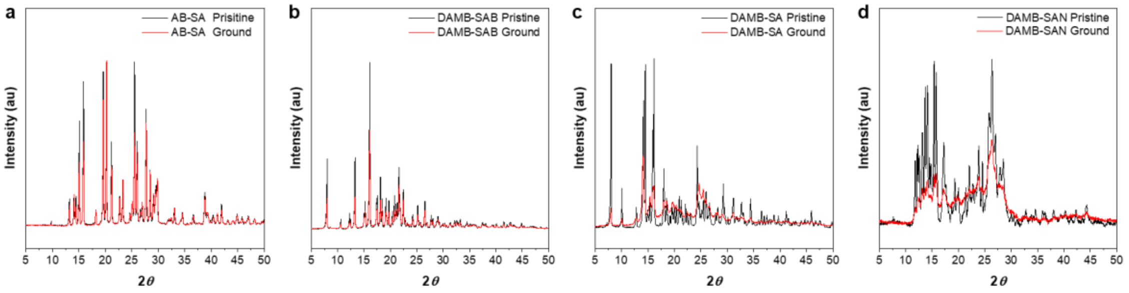

Supplementary Figure 24. PXRD patterns of (**a**) AB-SA, (**b**) DAMB-SAB, (**c**) DAMB-SA, and (**d**) DAMB-SAN as pristine and ground.

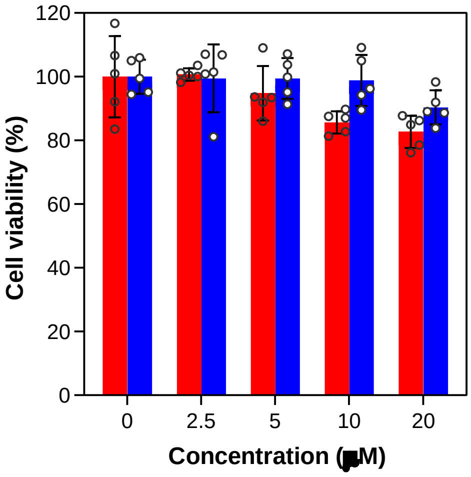

Supplementary Figure 25. Cell viabilities of COS-7 cells in the presence of different concentrations of DAMB-SA (red) and DAMB-SAN (blue), respectively. Data are presented as mean ± SD (n = 5 independent experiments).

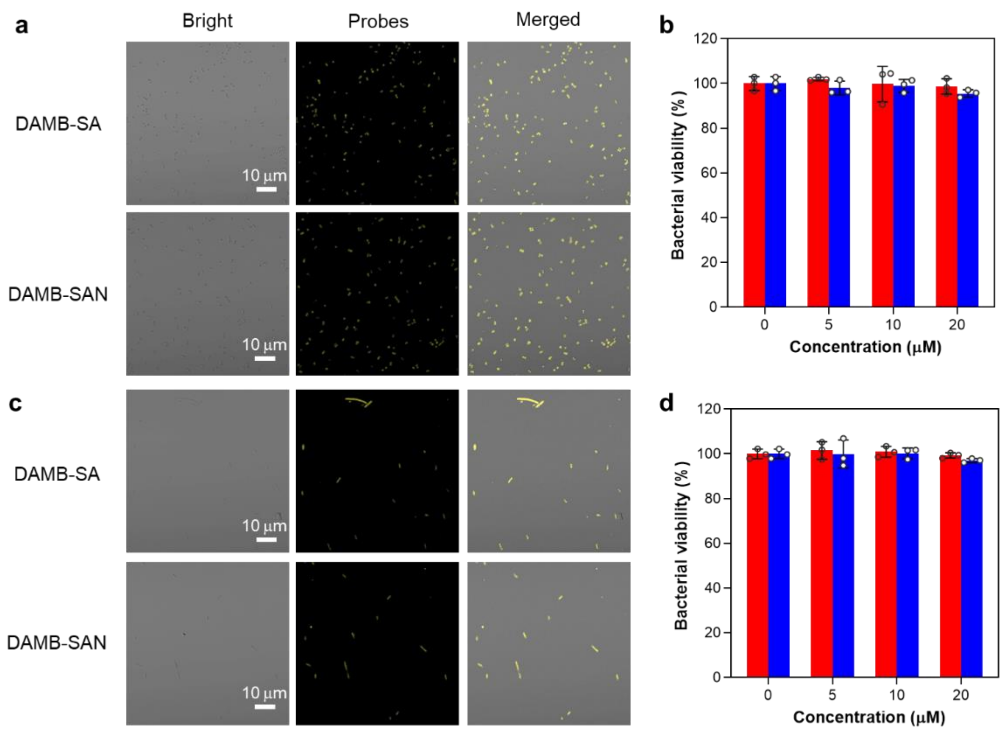

Supplementary Figure 26. a and c CLSM images of (**a**) *S. epidermidis* and (**c**) *E. coli* stained with 10 μM of DAMB-SA and DAMB-SAN for 30 min, respectively. λ_{ex}: 405 nm, λ_{em}: 540-600 nm. **b and d** Bacterial viabilities of (**b**) *S. epidermidis* and (**d**) *E. coli* in the presence of different concentrations of DAMB-SA (red) and DAMB-SAN (blue), respectively. Data are presented as mean ± SD (n = 3 independent experiments).

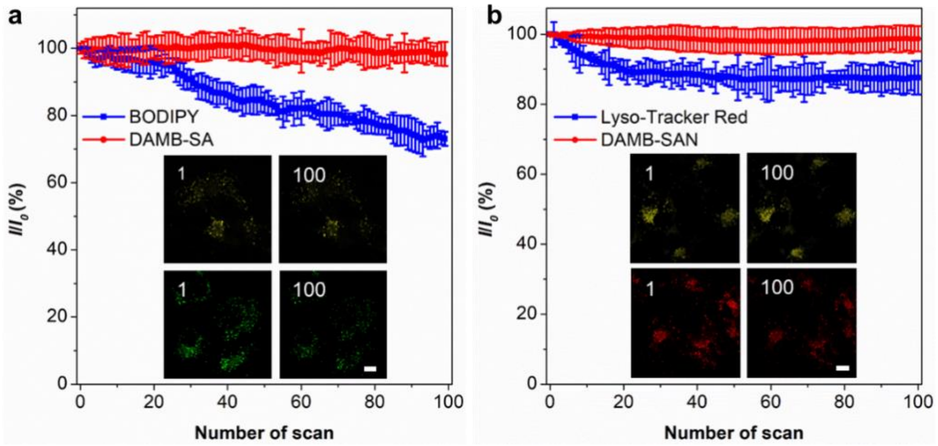

Supplementary Figure 27. Photostability of DAMB-SA (10 μM, yellow) and BODIPY (1 μM, green) (**a**), DAMB-SAN (10 μM, yellow) and Lyso-Tracker Red (1 μM, red) (**b**) in COS7 cells under continuous one-photon laser irradiation. DAMB-SA channel: λ_{ex}: 405 nm, λ_{em}: 540-600 nm; BODIPY channel: λ_{ex}: 488 nm, λ_{em}: 500-530 nm. DAMB-SAN channel: λ_{ex}: 405 nm, λ_{em}: 540-600 nm; Lyso-

Tracker Red channel: λ_{ex}: 543 nm, λ_{em}: 580-650 nm. Inset: Fluorescence images of COS7 cells with increasing number of scans. Data are presented as mean ± SD (n = 5 independent experiments).

References

1. Burr, A. H. & Hobson, A. D. The crystal and molecular structure of 2-bromo-N-salicylideneaniline. *Acta Cryst.* **B25**, 2662-2663 (1969).
2. Majorek, K. A. et al. Structural and immunologic characterization of bovine, horse, and rabbit serum albumins. *Mol. Immunol.* **52**, 174-182 (2012).
